# Clinical characteristics associated with future clozapine treatment among patients with bipolar disorder: A nationwide register-based study of 29,696 patients

**DOI:** 10.1192/j.eurpsy.2025.312

**Published:** 2025-08-26

**Authors:** E. Salagre, C. Rohde, S. D. Østergaard

**Affiliations:** 1 Department of Affective Disorders, Aarhus University Hospital; 2 Department of Clinical Medicine, Aarhus University, Aarhus, Denmark

## Abstract

**Introduction:**

Despite the number of medications considered as effective in the treatment of bipolar disorder (BD), incomplete response to treatment is very prevalent (Perlis *et al*. Am J Psychiatry 2006;163(2):217-24). Existing evidence supports the effectiveness of clozapine for treatment-resistant BD (TRBD) with clinical guidelines recommending clozapine as a third-line treatment (Yatham *et al.* Bipolar Disord. 2018;20(2):97-170). There is evidence to support that a shorter delay before clozapine initiation is associated with a better response to this treatment (Griffiths *et al.* Psychol. Med. 2021;51(3):376-86).

**Objectives:**

This study aimed to identify clinical and sociodemographic characteristics at the time of the first bipolar diagnosis associated with future clozapine treatment.

**Methods:**

We performed a population-based cohort study using nationwide data from Danish registries to investigate factors associated with initiation of clozapine treatment in incident BD. Cox proportional hazard regression analyses were used to investigate associations between patients’ characteristics at the time of the diagnosis of BD and a subsequent redemption of a prescription for clozapine, yielding hazard rate ratios (HRRs).

**Results:**

We identified a total of 29,696 patients registered with their first (incident) ICD-10 diagnosis of BD between 1999 and 2019, of whom 102 (0.3%) received clozapine treatment during follow-up. The median age at the first prescription of clozapine was 48.6 years (25-75 percentile: 37.7-59.9).  The multivariable Cox proportional hazards regression model showed that a prior diagnosis of psychotic disorder (other than schizophrenia or schizoaffective disorder) (HR: 2.10; CI: 1.13-3.93), having had three (HRR: 2.91; CI: 1.23-6.87), four (HRR: 2.89; CI: 1.15-7.24) or five or more (HRR: 3.17; CI: 1.19-8.44) previous psychopharmacological treatments prior to the diagnosis of BD, and being outside the labour force (HRR: 2.58; CI: 1.42-4.66) were positively associated with clozapine treatment after controlling for the remaining variables.

**Conclusions:**

The results of this study suggest that there are clinical characteristics associated with subsequent clozapine treatment already at the time of diagnosis of BD. These findings may guide targeted interventions, such as an earlier initiation of clozapine treatment.

**Disclosure of Interest:**

E. Salagre: None Declared, C. Rohde: None Declared, S. Østergaard Shareolder of: Dr. Østergaard reports ownership of units of mutual funds from companies with the stock tickers DKIGI, IAIMWC, SPIC25KL, and WEKAFKI; ownership/past ownership of units of exchange-traded funds with the stock tickers BATE, TRET, QDV5, QDVH, QDVE, SADM, IQQH, USPY, EXH2, 2B76, IS4S, OM3X, and EUNL during the conduct of the study, Grant / Research support from: Dr. Østergaard reported receiving the 2020 Lundbeck Foundation Young Investigator Prize; and grants from the Novo Nordisk Foundation (NNF20SA0062874), Lundbeck Foundation (R358-2020-2341 and R344-2020-1073), Danish Cancer Society (R283-A16461), Central Denmark Region Fund for Strengthening of Health Science (1-36-72-4-20), Danish Agency for Digitisation Investment Fund for New Technologies (2020-6720), and Independent Research Fund Denmark (7016-00048B and 2096-00055A) outside the submitted work

